# Innate and germline immune memory: specificity and heritability of the ancient immune mechanisms for adaptation and survival

**DOI:** 10.3389/fimmu.2024.1386578

**Published:** 2024-06-06

**Authors:** Diana Boraschi, Elfi Toepfer, Paola Italiani

**Affiliations:** ^1^ Shenzhen Institute of Advanced Technology, Chinese Academy of Sciences, Shenzhen University of Advanced Technology, Shenzhen, China; ^2^ Institute of Biomolecular Chemistry, National Research Council, Pozzuoli, Italy; ^3^ Stazione Zoologica Anton Dorhn, Napoli, Italy; ^4^ China-Italy Joint Laboratory of Pharmacobiotechnology for Medical Application, Shenzhen, China; ^5^ microfluidic ChipShop GmbH, Jena, Germany

**Keywords:** innate immunity, innate memory, specificity, adaptation, survival

## Abstract

The immune memory is one of the defensive strategies developed by both unicellular and multicellular organisms for ensuring their integrity and functionality. While the immune memory of the vertebrate adaptive immune system (based on somatic recombination) is antigen-specific, encompassing the generation of memory T and B cells that only recognize/react to a specific antigen epitope, the capacity of vertebrate innate cells to remember past events is a mostly non-specific mechanism of adaptation. This “innate memory” can be considered as germline-encoded because its effector tools (such as innate receptors) do not need somatic recombination for being active. Also, in several organisms the memory-related information is integrated in the genome of germline cells and can be transmitted to the progeny for several generations, but it can also be erased depending on the environmental conditions. Overall, depending on the organism, its environment and its living habits, innate immune memory appears to be a mechanism for achieving better protection and survival against repeated exposure to microbes/stressful agents present in the same environment or occurring in the same anatomical district, able to adapt to changes in the environmental cues. The anatomical and functional complexity of the organism and its lifespan drive the generation of different immune memory mechanisms, for optimal adaptation to changes in the living/environmental conditions. The concept of innate immunity being non-specific needs to be revisited, as a wealth of evidence suggests a significant degree of specificity both in the primary immune reaction and in the ensuing memory-like responses. This is clearly evident in invertebrate metazoans, in which distinct scenarios can be observed, with both non-specific (immune enhancement) or specific (immune priming) memory-like responses. In the case of mammals, there is evidence that some degree of specificity can be attained in different situations, for instance as organ-specific protection rather than microorganism-specific reaction. Thus, depending on the challenges and conditions, innate memory can be non-specific or specific, can be integrated in the germline and transmitted to the progeny or be short-lived, thereby representing an exceptionally plastic mechanism of defensive adaptation for ensuring individual and species survival.

## 1 Introduction

Immune memory is a well-studied phenomenon in mammals, and forms the basis for immune protection triggered by previous infections or vaccination. Exposure to microbial agents or their components can elicit an antigen-specific adaptive immune reaction targeting the triggering agent, encompassing production of neutralizing and opsonizing antibodies, generation of CD4^+^ and CD8^+^ cytotoxic T cells and other mechanisms (1). Typically, this response requires some time for developing (7–10 days), as the adaptive immune system should generate, by somatic recombination, specific tools uniquely recognizing and blocking the foreign agent (e.g., antibodies). Such adaptive immune response can be active for a period of time, as required for eliminating the danger, but it progressively wanes once the triggering agent is eliminated. This has become clear to everybody during the recent SARS-CoV-2 pandemic, as people received several vaccine jabs every few months, in order to sustain the anti-viral antibody levels, which otherwise would have disappeared. The protective immunity obtained by sustaining the immune response with repeated exposures to the antigen aims at achieving immediate inactivation of the infectious agent, when the pathogen is very rapidly infective and life-threatening.

Immune memory is a protective mechanism different from such sustained response (2). In mammals, upon elimination of the infective agent, the organism stops its immune activation (which is highly energy-demanding), and the immune system returns to baseline quiescence. However, memory cells generated by the encounter with the pathogen persist in certain niches in the body, and are already equipped to produce the required antigen-specific tools if/when the pathogen will come again (3, 4) Upon re-infection, such memory cells can mount a more efficient antigen-specific response in a much shorter time (1–2 days). The protective efficacy of immune memory depends on the type of pathogen. Infections with an incubation period of few days can be tackled effectively by immune memory cells, whereas pathogens that produce immediate damage (e.g., by producing toxins) can only be neutralized by a sustained response (e.g., pre-formed antibodies). Protective immunity and immune memory, both in response to infections and upon vaccination, are however more complex than a pathogen-specific response as described above, pertaining to the vertebrate/mammalian adaptive immunity.

The ancient evolutionarily conserved immune mechanisms of innate immunity are also primarily engaged in pathogen identification and elimination (5). Innate immunity is based on germline-encoded tools, such as the TLR receptors, and, because of this characteristic, is immediately ready to act against pathogens. For this reason, it was considered unable to “learn” and therefore to develop memory (6, 7). However, from evidence in plants and invertebrate metazoans, which only display germline-based immunity, as well as mammals, it is now clear that innate immunity can develop memory-like responses, based on different mechanisms and tools (e.g., metabolic and epigenetic changes that modulate and select subsequent gene expression, rather than somatic recombination that produces new effector molecules) (8–16). This memory-like immune reactivity is a mechanism of adaptation to the environment-derived cues (e.g., infectious agents), but it can be considered memory (which by the name must be specific and long lived) (2) because it can persist for the entire organism lifespan and be transmitted to the progeny, and it can also display a significant degree of specificity (see below). On this basis, we will define it as innate immune memory in the following paragraphs. The capacity of the innate immune memory responses to confer specific protection against pathogens is still a matter of investigation. Indeed, the dogma that innate immunity is fully non-specific (based on the lack of specificity of its recognition tools) is challenged by the abundant evidence in invertebrates and by accumulating evidence in mammals as well. We will discuss such evidence and propose a hypothesis on why and how innate immunity and its memory need and attain some degree of specificity despite its non-specific recognition tools.

## 2 Immune memory in invertebrates

Immune memory should be considered as a multidimensional concept, which is then declined differently in different organisms and with different underlying mechanisms (e.g., germline-encoded tools and epigenetic changes vs. somatic recombination mechanisms). The five key dimensions, as described by Pradeu and Du Pasquier (16), are: 1. strength, 2. duration, 3. speed, 4. specificity, and 5. extinction. Somatic recombination-dependent immune memory is in general characterized by high strength in the secondary response, a long duration, enhanced speed in the secondary response, high specificity and a clear extinction phase between the first and the second exposure. In organisms devoid of adaptive immunity (in particular in plants and invertebrate metazoans) the generation of memory-like immune adaptation is a well-known phenomenon, with organisms able to mount a more efficient immune response when re-exposed to a challenge (usually an infectious agent) (8, 9, 11, 15, 17). Memory-like immune reactivity in invertebrates has been described in at least three different forms, the recall response, the immune shift and the unique sustained response/acquired resistance (9, 16, 18–21). The recall response implies extinction of the immune response after the first exposure and a more effective response upon subsequent exposure. Notably, there is no evidence of an enhanced speed, most likely due to the fact that innate immunity is already very fast at the first challenge. This kind of memory-like response reflects the kinetic profile of adaptive immune memory in mammals, such as that induced by vaccination (22). The immune shift implies, after the first response, the generation upon re-exposure of a different and more protective kind of response, e.g., from phagocytosis to encapsulation (23, 24). It is interesting to note that also in this case there are similarities with adaptive immune memory, in which re-exposure induces a maturation of response, such as for instance the immunoglobulin class switch towards more efficient antibody classes (25, 26). The unique sustained response/acquired protection is probably not a memory response *sensu stricto*, since there is no extinction of the immune activation induced by the first exposure. Here, the response to a first challenge is kept at increasingly higher levels by re-exposure, achieving improved protection (27). The unique sustained response reflects the protective adaptive immunity attained in human beings by re-stimulation, e.g., with repeated vaccine boosts (28). The persistence of the antigen/agent that has triggered the response, even at low concentrations, is probably one of the elements at the basis of the persistent unique response in invertebrates, as well as of the sustained protective immunity in mammals. In this sense, the unique sustained response can be considered as an highly effective adaptation behavior, rather than a true memory response (2). However, since memory is linked to epigenetic reprogramming (both in animals and plants) (17, 29), the duration of immune memory is expected to depend on the persistence of the epigenetic changes underlying it, which in turn may depend on the presence of the memory-inducing agents/microorganisms in the living environment (30). In any case, other mechanisms may intervene in the establishment and maintenance of memory, including germline DNA modifications (see below). Thus, immune memory can last for the entire life of the organism, be maintained in daughter cells and transmitted to the progeny (9).

Specificity of the memory-like responses in invertebrates has been shown in several studies, mostly based on infection and re-infection of animals and assessment of the extent of secondary responses towards the original vs. unrelated microorganisms (31–40), although some studies might be biased by inadequate experimental design (16). Depending on the specificity of the secondary response, the invertebrate immune memory has been defined as immune enhancement (non-specific increase of secondary protective immunity) and immune priming (specific induction of improved secondary protective immunity) (see definitions in 20). The few examples below are meant to illustrate some of the many scenarios of specificity vs. selectivity of invertebrate immune memory.

In *Porifera* (sponges) and *Cnidaria* (corals), immune memory was described since the ‘80ies of last century as capacity of self/non-self-recognition in transplantation and parabiosis experiments (20, 41), leading to an accelerated specific rejection/destruction of a second allograft (42, 43). The rejection needs cell contact and is likely mediated by macrophage-like amoebocytic cells (41). Exposure of *Drosophila* to a sublethal dose of *Streptococcus pneumoniae* could induce a phagocyte-dependent life-long specific protection against a subsequent lethal challenge with the same microbe (36). Previous exposure of the oyster *Crassostrea gigas* to inactivated *Vibrio splendidus* bacteria could induce a potent secondary response to live *V. splendidus*, in terms of hemocyte recruitment and phagocytosis, but not to other *Vibrio* ssp. or unrelated bacteria (40). This memory-like immune adaptive response was at the level of the entire organism, as it depended on the specifically enhanced influx of hemocytes, while re-programming of cellular functions did not occur, as the phagocytic ability of individual hemocytes did not change. Conversely, in *Anopheles gambiae*, infection with *Plasmodium berghei* induced a protective memory recall response to *P. berghei* and also *P. falciparum*, which depended on the recruitment of the granulocytic effector cells to the infections site and their reprogramming towards enhanced parasite elimination (44). Another study in the terrestrial arthropod *Porcellio scaber* shows that *in vivo* infection with *Bacillus thuringiensis* strain 1 induced the subsequent increase of hemocyte phagocytic capacity against *B. thuringiensis 1* and *2*, but not *Escherichia coli*, a finding that suggests a re-programming at the cellular level (33). Hemocytes from the shrimp *Litopenaeus vannamei* exposed to the inactivated pathogen *V. harveyi* can develop an increased capacity to phagocytose the same bacteria, but not the unrelated *Bacillus subtilis*, upon a subsequent exposure (34). However, priming with *B. subtilis* could not induce a specific secondary increased phagocytosis, suggesting that memory induction was selective. In larvae of *Bombyx mori*, exposure to different bacteria raised a specific secondary phagocytic response, which could distinguish between Gram-positive and Gram-negative bacteria and also different strains within the same Gram-type. The memory response was both at the organism level (increased number of phagocytes and increased production of antibacterial factors) and at the cellular level (increased phagocytic capacity of individual cells). The memory response was also specifically protective against infection with live bacteria (35). Interestingly, an immune protective memory recall response was induced in the planaria *Schmidtea mediterranea* by exposure to the natural pathogen *Staphylococcus aureus*, but not by infection with *Legionella pneumophila* or *Mycobacterium avium*, which are not natural pathogens. The recall response was based on epigenetic changes and involvement of stem cells, which did not occur upon priming with non-natural pathogens (45). In the ascidian *Ciona robusta*, priming with gram-negative LPS or gram-positive LTA induced a memory recall response that largely depended on the secondary challenge. Irrespective of the priming agent, the recall response induced by an LPS challenge was a more potent cellular response (increased phagocytosis and expression of cellular receptors), while the recall response to LTA was a more potent humoral response (decreased phagocytosis, increase expression of complement components and cytokines) (19). In the crustacean *Artemia franciscana*, an abiotic stress (heat shock) could induce a protective secondary response both against subsequent heat shock and against infection with pathogenic *V. campbellii* (46). In *C. robusta*, exposure to abiotic stress (hypoxia + starvation) induces a recall response to bacterial LPS that differs between immune-competent organs: a general downregulation of cellular and humoral response genes in the pharynx and a general upregulation in the gut (47).

Thus, the immune memory-like adaptation responses in invertebrates can occur in different forms and adopt different degrees of recognition and specificity, but all aiming at affording better protection against recurrent pathogens or abiotic stress present in their living environment. Thus, a fully specific memory can be generated, for the targeted recognition and resistance to recurrent pathogens, which most likely are constantly present in the environment and provide the necessary level of re-boosting to afford a unique sustained response/acquired protection (33–35, 40). Also, memory seems to develop in response to natural pathogens but not upon priming with non-natural microorganisms (33, 44). The memory response can be challenge-specific, with priming just non-specifically making the immune system “ready” to adapt its memory response to the type of challenge (19). Eventually, the memory response induced by abiotic stress can be of broad specificity, affording a protective response against the specific agent but also against natural pathogens (46), and it can substantially differ in different immune-competent organs (47). The overall goal all these different immune memory-like forms is the successful adaptation to the environmental challenges, by developing a more protective response against recurrent specific threats.

While the mechanisms of memory responses are based on the same mechanisms of primary reactions (e.g., engagement of TLR and other innate receptors, RNA interference, phagocytosis, production of toxic molecules, *etc.*), the mechanisms by which memory is established and transmitted are not fully elucidated and can be different across evolution. Some of them will be described below (see paragraph 4.1. What drives the specificity and durability of innate immune memory?) and entail both the specificity of the memory responses and their heritability. Among the various mechanisms underlying the establishment of innate memory, it is worth mentioning endoreplication, *i.e.*, genome duplication in cells that do not undergo mitosis, probably as wider source of DNA when RNA and protein synthesis are required at very high levels (48). In mosquito and human cells, DNA duplication was observed as being essential for the upregulation of genes associated to trained immunity (such as phenoloxidase in mosquito cells and *TNFA* in human monocytes) upon priming with zymosan or *P. berghei* (mosquito) or β-glucan (human), while modulation of genes associated to tolerance, in monocytes primed with LPS, did not depend on DNA synthesis (49). This underlines the notion that innate memory is based on diverse mechanisms, which define the type, extent and duration of the memory reaction. These mechanisms can be either homologous across evolution, as in the above case of endoreplication underlying a potentiated memory response, or widely different, both across evolution and between different priming agents in the same organism.

## 3 Immune memory in mammals

In mammals, sophisticated specific immunological tools, such as antibodies and T and B cell receptors, have developed thanks to gene recombination and subsequent generation of diversity. The characteristics of adaptive or acquired immunity, as it is called, are the high specificity that allows precise recognition of very short epitopes, the capacity of developing a faster and more potent memory response, based on the persistence in a quiescent state of some T and B lymphocytes after a first specific immune response, and the long duration of the memory, which is stable as it based on gene recombination and not only on epigenetic changes.

Typically, it is thought that innate immunity has an initial protective role, having the advantage of being readily active (no need for gene rearrangement). Innate immunity can take care of the vast majority of invading microorganisms, as well as endogenous senescent, dying or transformed cells and misfolded/aggregated proteins (e.g., amyloid beta and tau protein aggregates in the brain), using a wide array of cellular effectors (in particular phagocytes such as macrophages and secretory cells such as mast cells) and soluble toxic mediators (in particular complement, reactive oxygen and nitrogen species and antimicrobial peptides). Adaptive immunity is involved at a later stage, only for tackling pathogens that could not be eliminated by innate immune mechanisms.

It is however clear that the two systems are not really separate in time and functions, and that they cross-talk constantly for coordinating the global protective response. Their functions are similar in that innate immune cells can also “learn” and react in a more effective fashion after having experienced an initial reaction, although the memory mechanisms of innate immunity are different from those of adaptive memory and their range and specificity of recognition is also different (broader in innate memory, highly specific in adaptive memory) and complementary. An innate immune memory, not based on gene recombination, is also present in mammals and has been reported, besides the abundant evidence in mice and humans, in other species including non-human primates, dogs and cattle (50–52), as well as in other vertebrates such as fish (53). The first well-documented descriptions of innate memory in mammals date back to the ‘40ies - ‘60ies of last century with the description of non-specific macrophage-dependent acquired resistance to infections in mice, in particular the seminal works of Paul Beeson (54), Rene Dubos (55) and the Youmans (56). *In vivo* and *in vitro* “priming” of mouse macrophages for a better secondary response was abundantly reported from the ‘70ies by several groups, including our own (e.g., 57–61), and was recently revamped and well developed by Netea group and dubbed “trained immunity” (13, 62, 63).

Innate immune memory is mainly based on epigenetic and metabolic changes (13, 14, 64, 65). Since epigenetic modifications can be transmitted to daughter cells and induced in hematopoietic progenitors in the bone marrow microenvironment, it is to be expected that innate memory can persist in tissues beyond the lifespan of the “primed” innate cells. However, epigenetic changes can also be rapidly eliminated by multiple mechanisms or replaced by different modifications upon repeated challenges. Thus, the memory of innate cells appears to be highly plastic, as it can constantly re-program the cellular functions in response to different environmental and microenvironmental cues, so as to rapidly adapt to the external and endogenous changes.

Several other mechanisms, in addition to epigenetic and metabolic reprogramming, are involved in the establishment of innate immune memory. For instance, RNA interference (see below) is a protective mechanism that appears to be maintained across evolution. A mechanism that affords short-term memory of previous inflammatory stimulation is the modulation of the NFκB dynamics (and consequent transcriptional response) through engagement of negative feedback modules, thereby inducing an adaptive response to new stimuli (66).

**Figure 1:**
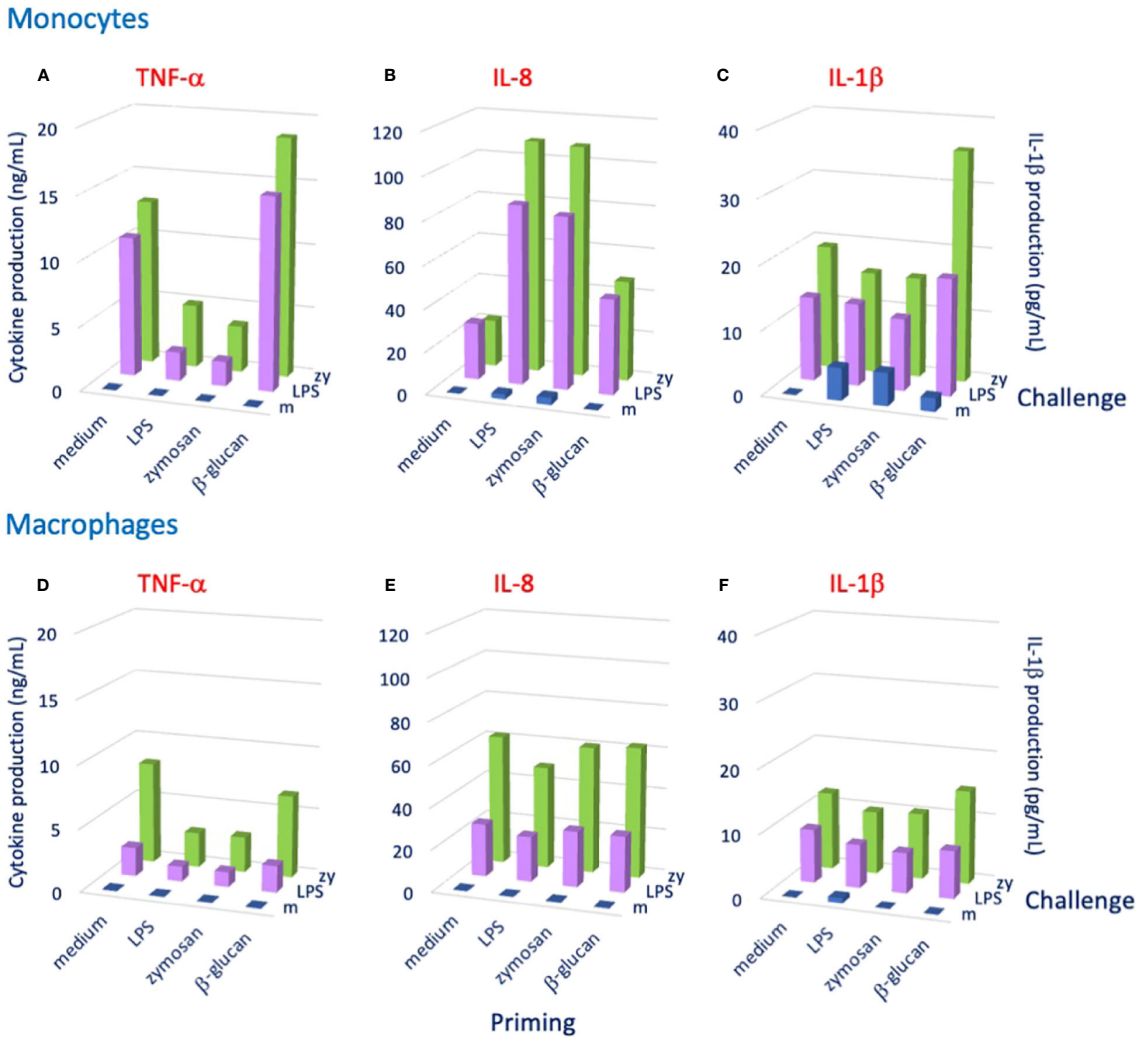
Human innate immune memory depends on both primary and secondary triggering agents. Human monocytes (panels **A–C**) or monocyte-derived macrophages (panels **D–F**) were primed for 24 h with culture medium alone (medium) or containing LPS (1 ng/mL), zymosan (1 μg/mL) or β-glucan (1 μg/mL) and, after 4 days of resting, challenged with medium alone (m), LPS (10 ng/mL) or zymosan (zy, 10 μg/mL). Cytokine production was measured after 24 h. Panels **(A, D)**: TNF-α; panels **(B, E)**: IL-8; panels **(C, F)**: IL-1β. Average data from 2–3 donors are shown. Full methodological description is provided in the **Supplementary Methods**, and full data are shown in the **Supplementary Table S1**.

Mammalian innate immune memory also appears to have some degree of specificity. The recognition tools of innate immunity, for instance TLR and scavenger receptors, are not highly specific as in the case of antibodies, but are nevertheless able to recognize different molecular patterns that characterize several classes of microorganisms. Different innate receptors can concomitantly bind different molecules on the same microorganism, thereby establishing a strong multiple interaction that blocks the target and initiates its destruction. Thus, specificity of innate immune cells is in principle determined by the suite of receptors engaged in the interaction. The lack of recombination and generation of diversity, typical of adaptive immunity, is replaced by the abundance of pre-formed germline receptors and by multiple concomitant recognition by such receptors, as in the case of innate immunity in invertebrates (6, 7, 67). Also, NFκB-based memory responses are different depending on the identity and dose of the priming stimulus (66). The features of the initial recognition (e.g., the suite of engaged receptors and triggered signaling pathways) are likely responsible for the types of epigenetic and metabolic changes that will determine the features of a secondary memory response. Thus, priming with LPS typically induces a “tolerance” type of memory response (less production of inflammatory factors), while priming with β-glucan or BCG induces a potentiated memory response (63, 68), probably exploiting the persistence of the priming agents (BCG can survive 1–2 months within macrophages, and β-glucan is hardly degraded after being phagocytosed by macrophages) (2). Notably, as also observed in invertebrates, the changes induced by priming agents do not determine a particular type of non-specific secondary response (tolerance or immunoenhancement), but rather set the basis for a more specific response that depends on the challenging agent. An example is provided in **Figure 1**, showing the memory response of human monocytes primed in culture with different agents (LPS, the zymosan yeast particles and β-glucan) and later challenged with LPS or zymosan. The memory response, measured as production of three different inflammation-related factors, shows that priming with LPS or zymosan (made of insoluble β-glucan) induced an identical secondary response (decreased TNF-α, increased IL-8, unchanged IL-1β) independently of challenge, whereas priming with β-glucan induced a memory response that depends on the challenge (tendency to increased TNF-α and IL-8 and unchanged IL-1β in response to an LPS challenge; tendency to increased TNF-α and IL-8 and a substantial increase of IL-1β in response to a zymosan challenge) (**Figures 1A-C**). Thus, a challenge with zymosan specifically induces an enhanced response in terms of IL-1β production, whereas the potentiated response in terms of TNF-α and IL-8 production seems to be less evident and non-specific. It is notable that the same substance (β-glucan) can induce different types of memory depending on its form (insoluble zymosan particles vs. soluble molecule), suggesting that the induction of memory is modulated by the size/solubility of the priming agent. It is also important to note that the same combination of priming and challenge can generate a different memory response in monocyte-derived macrophages. Priming with LPS or zymosan induced a tolerance response to LPS or zymosan in terms of TNF-α production, but no significant change in IL-8 and IL-1β (**Figures 1D-F**). Eventually, priming with β-glucan induced no change in any of the three cytokines in response to either LPS or zymosan (**Figures 1D-F**). Thus, each particular combination of priming and challenging stimuli can induce a specific memory response profile, which can also differ between different innate cell types. Thus, the innate memory response is in its way antigen-specific, as it is based on the cell-specific and priming/challenge-specific combined increased or decreased production of a range of inflammatory/immunostimulatory and anti-inflammatory/immunomodulatory factors that is expected to optimally support a defensive reaction to the new challenge.

Another notable feature of innate memory specificity in mammalians relates to organ tropism. There is evidence both in mice and humans that exposure to lung infectious agents can induce an innate memory that will protect against the same or other lung-specific infections or diseases (e.g., asthma), but not, or less potently, against infections/allergies typical of other body compartments (69–72). Although this is not always the case, and systemic immune memory can be generated after priming in different anatomical compartments, this suggests that the organ-specific microenvironment can play a role in the establishment and maintenance of topically protective innate memory.

## 4 Discussion and conclusions

### 4.1 What drives the specificity and durability of innate immune memory?

While there is abundant evidence on the role of epigenetic and metabolic changes as the basis for the cellular re-programming underlying innate memory generation and maintenance, it is much less clear how its specificity is established and how it can be transmitted transgenerationally. From the experimental evidence accumulated in recent years, it is clear that innate immune memory is an adaptive mechanism to better cope with repeated challenges coming from the external environment and, in mammals, also from the organ-specific microenvironment. In invertebrate metazoans and in non-metazoan organisms, several highly effective memory-like protective mechanisms can be observed, besides the epigenetic and metabolic changes described for mammalian innate memory. A notable example is the RNA-based memory, which illustrates the convergent evolution, in different taxa, of distinct and often parallel mechanisms that aim to reach the same objective (protection against infections). In *Archaea* and *Bacteria*, the CRISPR-Cas system provides the memory of past infections (integration of the invaders’ sequences into the CRISPR locus) and the capacity to generate small interfering RNAs (crRNAs) for silencing the next infection, with the help of a large array of Cas proteins (which can also be transmitted horizontally to enlarge their availability) that contribute to synthesis and specific targeting of the crRNAs (73). In insects, and mosquitoes in particular, the best-known immune defensive mechanism against viruses is RNA interference (RNAi) by small interfering RNAs and PIWI-interacting RNAs (piRNAs) (74, 75). Endogenous viral sequences integrated into the insect genome can function as template for the production of piRNAs (76, 77) and also act as an archive of past viral infections. The genome loci rich in such sequences are activated by viral infection to produce specific piRNAs that restricts replication of the infecting virus (78, 79). These mechanisms of “memory” of past infections resembles the high specificity of mammalian adaptive immunity, although not based on somatic recombination for the generation of such specificity. Other examples of protective memory involving RNAi are those observed in the helminth *Caenorhabditis elegans*, in which double stranded RNA (dsRNA) could induce RNAi across the tissues and from soma to germline (80) through the production of endogenous siRNAs that are shuttled to the nucleus where they regulate target gene transcription (81–83). Heritable siRNAs are carried to the germline by Argonaute proteins, with different proteins binding different populations of small RNAs and promoting different gene regulatory effects (83–85). RNAi heritability may last for many generations or be lost after 3–4 generations, as it is modulated by heritable small endoRNAs, whose production is affected by the original dsRNA-induced RNAi and is sensitive to environmental stress, and that regulate the RNAi pathway (86, 87). It is hypothesized that this active tuning of memory heritability is an adaptive mechanism that allows for maintaining or eliminating the RNAi memory to better adapt to changes of environmental conditions and avoid possible detrimental effects (87). Another example of RNAi-based protective memory is the avoidance behavior of *C. elegans* that develops after a first encounter with the pathogen *Pseudomonas aeruginosa* (88). Memory is due to the upregulation of an avoidance protein that involves recognition of small bacterial RNAs, activation of RNAi pathway, piRNA regulation in the germline and eventual downregulation of a worm gene homologous to the bacterial small RNA, which is a regulator of the avoidance gene (89). The avoidance memory can be transferred to the progeny for several generations by the activity of the *Cer1* retrotransposon, which conveys the avoidance information from germline to somatic cells (90). It is notable that the protective memory based on RNA interference is displayed by different cells (the unicellular organisms in *Archaea* and *Bacteria*, any infected cell in invertebrates, including sensory neurons in *C. elegans*), stressing the concept that protective memory is “immune” in a much wider sense than the activation of cells of the immune system. Besides non-metazoan organisms and metazoan invertebrates, RNAi, with both common and taxon-specific mechanisms, appears to be a major antimicrobial immune defense in all eukaryotic organisms, from plants to mammals (91).

Maintaining the epigenetic changes induced by exposure to infectious agents, therefore, seems to depend on several factors. As mentioned above, the integration of microbial sequences in the host genome could stably maintain the pathogen-related information necessary for a targeted defense and transfer it across generations despite the reprogramming of the germline (92). Another factor is the re-exposure to infectious agents commonly present in the living environment, as in the case of many invertebrates that live their entire life in the same place. In this case, the most common immune memory type is a unique sustained response/acquired resistance, which maintains a life-long high protection level. In mammals and humans, the living habits are different, and include moving across different environments and increased chance of encounters with new pathogens. This may be one of the reasons for “upgrading” the immune response from innate to adaptive, with the generation of endless recombination-generated specific immune effectors to help innate immunity to cope with unexpected challenges. Innate immune memory is still established, with a duration in time and a degree of selectivity/specificity that depend on the type of sequential exposures. In addition, the anatomical complexity of many invertebrates and vertebrates is possibly a reason for an organ-specific type of innate immune memory, which can be maintained by the organ microenvironmental cues and that can protect against several types of organ-tropic challenges. Transgenerational transmission of innate memory can occur when the changes that have been induced in the genome of somatic cells (e.g., integration of viral-derived sequences) are transferred from soma to germline, as it happens in *C. elegans* and in other invertebrates. However, there are tunable mechanisms that can maintain or erase the transgenerational transfer of memory, in order to afford optimal protection and less detrimental effects upon changes in the environmental conditions. Thus, heritable memory can last for several generation but disappear if/when such memory may become detrimental rather than advantageous. In mammals, there is some evidence of transmission of innate memory to the progeny in the mouse, with male mice infected with *C. albicans* or treated with zymosan able to transmit heterologous resistance to infections to the next generation, allegedly due to introduction of infection-induced epigenetic changes (DNA methylations) in the sperm of infected males during spermatogenesis (93). It is however unknown whether these DNA methylations in sperm DNA may be maintained across generations (transgenerational transmission) or whether the infection during spermatogenesis is needed for inducing them (intergenerational transmission). Notably, another accurate study, using BCG as inducer of innate memory in mice, showed that protective innate memory cannot be transmitted to the progeny (94), and suggests that, in placental lactating animals, vertical transmission of innate protective mechanisms may have been largely replaced by mechanisms of adaptive immunity, such as antibody transfer.

In summary, protective memory has developed, across evolution, different mechanisms that can ensure specificity, lifelong protection, horizontal and vertical transmission, but that can be shut off or tuned, to adapt to the changes in the environmental conditions and cues. In vertebrates, the presence of the highly specific adaptive immune memory, based on somatic recombination, may have replaced the specificity largely displayed by innate immunity and memory in invertebrates. Conversely, some protective memory mechanisms, such as RNA interference, seem to be maintained across evolution. A summary of the homologies/analogies and differences in the specificity and heritability of innate immune memory across evolution is presented in **Table 1**.

**Table 1:** Specificity and heritability of innate immune memory across evolution.

SPECIFICITY AND HERITABILITY OF INNATE IMMUNE MEMORY				
Taxon	Characteristics	Innate defensive strategies and tools entailing specificity	Innate memory transferability	Ref
Prokaryotes *Archaea* and *Bacteria*	• Unicellular • Adapted to restricted environments	• CRISPR-Cas system (specific) • RNA interference (specific) • Intracellular pattern recognition receptors (selectivity)	• Horizontal gene and protein transfer • Vertical gene transfer	Reviewed in (73)
Eukaryotes Plants	• No mobility • No specialized immune system • High chance of re-encountering pathogens living in the same environment	• Pattern recognition receptors (selectivity) • Production of soluble and gaseous defensive mediators and enzymes (non-specific) for systemic immunity • Physical isolation of invaders (non-specific) • Histone modifications and chromatin remodeling (non-specific)	• Transfer to other body parts (from local to systemic resistance) • Gaseous transfer to neighbouring plants • Transfer of resistance to progeny (DNA methylation, others) • Transfer of susceptibility to priming (primed-to-be-primed phenotype)	Reviewed in (17)
Eukaryotes Invertebrates	• Limited mobility across habitats • Endowed with innate immunity • Lack of somatic-recombination-based immunity • High chance of re-encountering pathogens living in the same environment	• Specialized immune cells • Multiple pattern recognition receptors (improved selectivity) • Antimicrobial molecules (non-specific) • Enzymes (non-specific) • Phagocytosis and intracellular elimination • Physical isolation of invaders (e.g., encapsulation & melanization; non-specific) • RNA interference (specific)	• Horizontal transfer (e.g., via virus-like particles) • Intergenerational germline transfer • Transgenerational germline transfer	Reviewed in (8, 9, 11, 18, 20, 21, 31)
Eukaryotes Vertebrates	• Endowed with both innate and somatic recombination-based immunity • Anatomical complexity • Extensive mobility across habitats	• Specialized immune cells • Pattern recognition receptors (selectivity) • Production of soluble defensive and toxic mediators (non-specific) • Phagocytosis and intracellular elimination • Anatomical diversification of cells and mediators (selectivity) • Physical isolation of invaders (*e.g.*, granulomas; non-specific) • RNA interference • NFκB dynamics	• Intergenerational transfer in mice (unconfirmed)	Reviewed in (2, 5–7, 13–15, 22, 28, 57–59, 61–72, 91, 93, 94)

### 4.2 Do we need a new nomenclature for immune responses and immune memory?

Classically, we define immunity in mammals in a dicotomic fashion, as composed by two different systems, the adaptive or acquired immunity, *i.e.*, the immunity that can learn and have memory and is highly specific, and the innate immunity, non-specific, always active at the same level and that does not develop memory. Now we know that innate immunity can learn, adapt, develop memory and be specific, and this happens not only in invertebrates but also in mammals. Thus, some terms that have been used for many years by immunologists may currently generate confusion and misunderstanding, in light of more recent knowledge. For instance, in this paper, we have used the term “adaptive” for defining the mammalian adaptive immunity (T and B cells, antibodies), but also for illustrating the capacity of innate immunity to adapt to the environmental conditions. Also, a common synonym used for adaptive immunity is “acquired immunity” (to underline the acquisition of specificity upon a first exposure), but a type of invertebrate innate memory has been named “acquired resistance”. The confounding nomenclature used by immunologists active in different areas was very well pointed out and emphasized by Pradeu and Du Pasquier in their excellent essay on immunological memory (16), and urges the scientific community to update the terminology. Here, we have used the term “innate immune memory” to encompass all the various phenomena of the protective memory that is not based on somatic recombination (*i.e.*, excluding adaptive immune memory in vertebrates): these phenomena have been defined as immune priming, immune enhancement, recall response, immune shift, unique sustained response, trained immunity, tolerance. “Innate immune memory” may sound self-contradictory (innate memory means a pre-existing memory, which is not what we want to say), but we intend the term “innate” as opposite of “somatic-recombination-based” (which is the case of adaptive immune memory). Even the term “memory” may be misleading, because memory is expected to be long-term, which is not always the case for innate responses (2), although there is evidence that protective memory not only can last life-long but it can even be passed to the progeny, as a real germline-encoded memory. Since we have become aware that memory is a flexible concept, with different duration depending on the external and endogenous scenarios (even when pertaining neurological memory) we think that the term fits well the phenomenon if accompanied by “innate” and “immune”. The term “trained immunity”, which is well accepted and widely used by human immunologists, intends to define a specific aspect of innate memory (the enhancement of the secondary response), but it is a term that cannot really distinguish between the memory of innate immunity and that of adaptive immunity, since both need “training” for learning and improving. The same goes for the terms adaptation, priming, enhancement, immunoimprinting, which describe the phenomenon well, but again cannot distinguish between germline and somatic recombination-dependent responses. Indeed, the development of innate immune memory implies adaptation to the upcoming challenges and acquisition of better protective tools, the same as in the mammalian somatic recombination-dependent immune memory, although with different mechanisms. We would therefore encourage the immunology community to re-consider the nomenclature of the immune system so that the names could better reflect the functions.

## Data availability statement

The original contributions presented in the study are included in the article/**Supplementary Material**. Further inquiries can be directed to the corresponding author.

## Ethics statement

The studies involving humans were approved by Regional Ethics Committee for Clinical Experimentation of the Tuscany Region (Ethics Committee Register n.14,914 of 16 May 2019). The studies were conducted in accordance with the local legislation and institutional requirements. The participants provided their written informed consent to participate in this study.

## Author contributions

DB: Conceptualization, Data curation, Funding acquisition, Writing – original draft, Writing – review & editing. ET: Investigation, Methodology, Writing – review & editing. PI: Conceptualization, Data curation, Investigation, Methodology, Writing – review & editing.
