# Supplementary material for: Innate and germline immune memory: specificity and heritability of the ancient immune mechanisms for adaptation and survival
Source: Front Immunol. 2024 Jun 6;15:1386578. doi: 10.3389/fimmu.2024.1386578 (PMC11186993; doi:10.3389/fimmu.2024.1386578)
Supplement: Supplementary file 1 [file Table_1.docx]

Supplementary Material

Innate and germline immune memory: specificity and heritability of the ancient immune mechanisms for adaptation and survival

Diana Boraschi*, Elfi Toepfer and Paola Italiani

*** Correspondence:** Diana Boraschi: diana.boraschi@gmail.com

# Supplementary Methods

*Monocyte isolation and macrophage differentiation*

Blood was obtained from healthy donors after obtaining informed consent, in keeping with the Declaration of Helsinki. The ethical approval of the protocol was obtained from the Regional Ethics Committee for Clinical Experimentation of the Tuscany Region (Ethics Committee Register n. 14,914 of 16 May 2019).

Peripheral blood mononuclear cells (PBMC) were obtained by Ficoll-Paque gradient density separation (GE Healthcare, Bio-Sciences AB, Uppsala, Sweden). Monocytes were isolated from PBMC by CD14 positive selection with magnetic microbeads (Miltenyi Biotec, Bergisch Gladbach, Germany). Purity and viability of monocyte preparations were assessed by trypan blue exclusion and cytosmears, respectively, and confirmed to be > 95%.

Monocytes were seeded at 5x10^5^ cells/mL in wells of 24-well plates (Corning® Costar®; Corning Inc. Life Sciences, Oneonta, NY, USA) in 1 mL of culture medium (RPMI 1640 + Glutamax-I; GIBCO by Life Technologies, Paisley, UK) supplemented with 50 μg/mL gentamicin sulfate (GIBCO) and 5% heat-inactivated human AB serum (Merck Sigma-Aldrich®, St. Louis, MO, USA) and kept overnight at 37ºC in moist air with 5% CO_2_, before starting the experiments.

For macrophage differentiation, blood monocytes, isolated and characterized as described above, were cultured in complete medium supplemented with 50 ng/mL human recombinant CSF-1 (R&D Systems, Minneapolis, MN, USA) for 6 days (with one medium change after 3 days).

With these isolation and culture protocols, monocytes were CD14^+^ CD64^+^ CX3CR1^+^ CD206^-^ and CD80^-^, whereas monocyte-derived macrophages were heterogeneous for CD14 and CD64 expression (about 50% positive), but all CX3CR1^+^ CD206^+^ and CD80^-^ by flow cytometry (1).

*Innate memory responses*

For assessing the memory responses, monocytes and macrophages were first exposed for 24 h to culture medium alone (medium/negative control) or containing 1 ng/mL LPS (positive control; from *E. coli* O55:B5; Merck Sigma-Aldrich®), 1 μg/mL zymosan (insoluble β-1,3-glucan polysaccharidic particles from *Saccharomyces cerevisiae*; InvivoGen, San Diego, CA, USA), or 1 μg/mL β-glucan (extracted from *Candida albicans*; a generous gift from Charles Dinarello, University of Colorado, Denver, CO, USA). Supernatants were collected and assessed by ELISA for inflammatory cytokines, to confirming cell activation (data not shown). Cells were then washed and cultured with fresh culture medium for 4 additional days. Return to baseline (*i.e.*, completion of the extinction phase) was determined by the absence of inflammation-related cytokines in the supernatant. After the resting phase, cells were challenged for 24 h with fresh medium alone or containing 10 ng/mL LPS or 10 μg/mL zymosan (*i.e.*, a 10x higher concentration than in the primary stimulation, to reproduce a stronger secondary exposure). Supernatants were collected for cytokine evaluation.

Visual inspection confirmed that cell viability and cell number did not substantially change between different treatments.

*Evaluation of Cytokine Production*

The human inflammatory cytokines TNFα and IL-1β and the chemokine IL-8, produced by monocytes and macrophages and released in the cell culture supernatants, were measured by ELISA (R&D Systems) with a Cytation 3 imaging multi-mode reader (BioTek, Winooski, VT, USA). Each sample was tested in duplicate in ELISA.

*Statistical Analysis*

TNFα and IL-8 data are expressed as ng of produced cytokine/mL (corresponding to 5x10^5^ input cells), while IL-1β data are reported as pg/mL. Results are presented as mean ± SEM of data from 2-3 replicate donors. From each donor, both monocytes and monocyte-derived macrophages were obtained. The statistical significance of differences is indicated by *p* values, which were calculated with unpaired and two-tailed Student’s *t*-tests.

*References*

1. Madei MP, Toepfer E, Boraschi D, Italiani P. Different regulation of interleukin-1 production and activity in monocytes and macrophages: innate memory as an endogenous mechanism of IL-1 inhibition. Front Pharmacol (2017) 8: 335. doi: 10.2289/fpharm.2017.00335

# Supplementary Table S1: Innate memory response of stimulus-primed monocytes and macrophages challenges with LPS or zymosan

| **TNF-α** | Cytokine production (mean ± SEM ng/mL) | | | | |
| --- | --- | --- | --- | --- | --- |
| **MONOCYTES** | ***Challenge*** | | | | |
| ***Priming*** | *medium* | | *LPS* | *zymosan* | |
| medium | 0.00 ± 0.00 | | 10.88 ± 1.10 | 12.86 ± 17.16 | |
| LPS | 0.01 ± 0.01 | | 2.26 ± 0.60* | 4.92 ± 1.11* | |
| zymosan | 0.01 ± 0.00 | | 1.94 ± 0.80** | 3.62 ± 1.08** | |
| β-glucan | 0.02 ± 0.02 | | 14.98 ± 2.76 | 18.63 ± 3.25 | |
| **MACROPHAGES** | ***Challenge*** | | | | |
| ***Priming*** | *medium* | | *LPS* | *zymosan* | |
| medium | 0.05 ± 0.01 | | 2.28 ± 0.47 | 8.07 ± 1.69 | |
| LPS | 0.06 ± 0.00 | | 1.20 ± 0.22 | 2.77 ± 0.62** | |
| zymosan | 0.04 ± 0.01 | | 1.17 ± 0.26 | 2.82 ± 0.67** | |
| β-glucan | 0.05 ± 0.01 | | 2.13 ± 0.38 | 6.54 ± 0.62 | |
| **IL-8** | Cytokine production (mean ± SEM ng/mL) | | | | |
| **MONOCYTES** | ***Challenge*** | | | | |
| ***Priming*** | *medium* | | *LPS* | *zymosan* | |
| medium | 0.14 ± 0.02 | | 26.71 ± 15.42 | 21.95 ± 12.67 | |
| LPS | 2.31 ± 1.60 | | 84.00 ± 22.09 | 108.89 ± 26.59 | |
| zymosan | 3.35 ± 2.37 | | 80.48 ± 30.28 | 107.79 ± 43.69 | |
| β-glucan | 0.16 ± 0.03 | | 49.84 ± 11.12 | 52.38 ± 11.65 | |
| **MACROPHAGES** | ***Challenge*** | | | | |
| ***Priming*** | *medium* | | *LPS* | *zymosan* | |
| medium | 0.26 ± 0.04 | | 25.27 ± 1.43 | 61.86 ± 10.83 | |
| LPS | 0.22 ± 0.05 | | 21.68 ± 2.93 | 48.84 ± 8.00 | |
| zymosan | 0.35 ± 0.11 | | 26.62 ± 3.47 | 60.58 ± 10.64 | |
| β-glucan | 0.21 ± 0.03 | | 26.73 ± 2.09 | 62.48 ± 11.79 | |
| **IL-1β** | Cytokine production (mean ± SEM pg/mL) | | | | |
| **MONOCYTES** | ***Challenge*** | | | | |
| ***Priming*** | *medium* | *LPS* | | | *zymosan* |
| medium | 0.00 ± 0.00 | 13.08 ± 3.54 | | | 19.16 ± 4.45 |
| LPS | 5.07 ± 1.52 | 12.76 ± 1.03 | | | 15.69 ± 1.11 |
| zymosan | 5.15 ± 1.54 | 11.21 ± 0.85 | | | 15.54 ± 2.03 |
| β-glucan | 2.14 ± 2.14 | 18.07 ± 5.04 | | | 35.61 ± 12.13 |
| **MACROPHAGES** | ***Challenge*** | | | | |
| ***Priming*** | *medium* | *LPS* | | | *zymosan* |
| medium | 0.00 ± 0.00 | 8.42 ± 2.74 | | | 12.20 ± 0.00 |
| LPS | 0.76 ± 0.70 | 6.83 ± 0.97 | | | 9.98 ± 1.05 |
| zymosan | 0.00 ± 0.00 | 6.34 ± 2.61 | | | 10.34 ± 0.93 |
| β-glucan | 0.00 ± 0.00 | 7.47 ± 2.42 | | | 14.68 ± 3.60 |

Data are the mean ± SEM of cytokine levels (ng/mL) assessed in 3-7 replicate determinations on cells from 2-3 individual donors. A tendency to increase can be observed for IL-8 production by monocytes primed with LPS or zymosan and for IL-1β upon priming with β-glucan and challenge with zymosan. Such increases were observed in each individual donor, although their means are not significantly different from controls, due to the large donor-to-donor variability in the levels of cytokine production. **p*<0.05; ***p*<0.005
